# RNAseq of diverse spring wheat cultivars released during last 110 years

**DOI:** 10.1038/s41597-023-02769-w

**Published:** 2023-12-08

**Authors:** Saman Maqbool, Samar Naseer, Nageen Zahra, Fatima Rasool, Humaira Qayyum, Khawar Majeed, Muhammad Jahanzaib, Muhammad Sajjad, Muhammad Fayyaz, Muhammad Kashif Naeem, Muhammad Ramzan Khan, Hao Zhang, Awais Rasheed, Huihui Li

**Affiliations:** 1 State Key Laboratory of Crop Gene Resources and Breeding, Institute of Crop Sciences, Chinese Academy of Agricultural Sciences (CAAS), CIMMYT-China office, Beijing, China; 2 Department of Plant Sciences, Quaid-i-Azam University, Islamabad, 45320 Pakistan; 3 National Institute of Genomics and Advanced Biotechnology (NIGAB), National Agriculture Research Center (NARC), Islamabad, Pakistan; 4 Oilseeds Research Program, Crop Sciences Institute (CSI), National Agriculture Research Center (NARC), Islamabad, Pakistan; 5 Department of BioSciences, COMSATS University, Islamabad, Pakistan; 6 Crop Disease Research Institute, National Agriculture Research Center (NARC), Islamabad, Pakistan; 7 Nanfan Research Institute, CAAS, Sanya, Hainan China

**Keywords:** Plant breeding, Plant genetics

## Abstract

Here, we performed RNA-seq based expression analysis of root and leaf tissues of a set of 24 historical spring wheat cultivars representing 110 years of temporal genetic variations. This huge 130 tissues RNAseq dataset was initially used to study expression pattern of 97 genes regulating root growth and development in wheat. Root system architecture (RSA) is an important target for breeding stress-resilient and high-yielding wheat cultivars under climatic fluctuations. However, root transcriptome analysis is usually obscured due to challenges in root research due to their below ground presence. We also validated the dataset by performing correlation analysis between expression of RSA related genes in roots and leaves with 25 root traits analyzed under varying moisture conditions and 10 yield-related traits. The Pearson’s correlation coefficients between root phenotypes and expression of root-specific genes varied from −0.72 to 0.78, and strong correlations with genes such as *DRO1*, *TaMOR*, *ARF4*, *PIN1* was observed. The presented datasets have multiple uses such as a) studying the change in expression pattern of genes during time, b) differential expression of genes in two very important tissues of wheat i.e., leaf and roots, and c) studying customized expression of genes associated with important phenotypes in diverse wheat cultivars. The initial findings presented here provided key insights into understanding the transcriptomic basis of phenotypic variability of RSA in wheat cultivars.

## Background & Summary

Bread wheat (*Triticum aestivum*) is one of the most important staple food crops providing 55% of carbohydrates to the world population. The grain yield of wheat has to increase at an average annual rate of ~2% in a limited area of cultivated land to meet the world food demand^1^. A deeper understanding of wheat genetics is required to address the primary challenge of sustaining food security in the context of climate change to feed the growing population. It is critical to deepen the knowledge of the wheat genomics and its genetic composition as well as the broad range of sequencing and transcriptomics data to understand genetic basis of wheat adaptability to target environments^2^. Identification and functional characterization of genes that regulate developmental stages critical for withstanding climatic fluctuations is an important aspect of this area of research. Similarly, it is central to functional genetic studies to analyze dynamic expression patterns of each gene contributing to plant development in various tissues and response to various environmental stimuli^3^.

Roots are significant for the production of food grains such as wheat and rice^4^. A variety of morphological and physiological traits expressed by root systems facilitate the uptake of water and nutrients. Similar to above-ground traits, there must be an understanding of unique root system architecture (RSA) for optimum resource acquisition^5^. Since roots are important components of breeding programs, it is crucial to understand the molecular mechanisms involved in root formation especially under challenging conditions.

In order to pinpoint the genetic components influencing the root growth in maize, rice and other crops, a variety of forward and reverse genetics techniques including transcriptomics and functional genomics have been applied^6^. The transcriptome studies using next-generation sequencing (NGS) technologies have paved the way in linking genotype to phenotype and can detect the molecular mechanisms underlying plant responses to abiotic stress^7^. Presently, several population-wide transcriptome analyses have been conducted in cereal crops including rice^8^, wheat^9^, and barley^10^. These studies unravelled the associations between gene expression and traits; however, field studies have generally been restricted to transcriptomics of above-ground shoots due to the challenge of sampling root tissues in field conditions. RNA-seq previously known as whole transcriptome shotgun sequencing has excitingly shaped whole transcriptome profiling^7^. It can identify transcript levels, expressed polymorphisms, and splicing isoforms. The development of high-throughput next-generation RNA-seq technologies provides new insights into transcriptome analysis such as a detailed expression profile, higher sensitivity to genes expressing at both high and low extremes, and no limitation by the lack of prior genome knowledge^11^. RNA-seq studies in wheat are increasing rapidly owing to the reconstruction of the entire transcriptome using the short paired-end (PE) assembly of de novo reads^12^ and provide a precise measurement of transcript levels. In wheat, some large-scale RNAseq studies available where transcriptome of multiple tissues from a single cultivar are reported like in Chinese Spring and Azhurnaya^13^. In this study, we conducted transcriptome profiling using RNA-seq on a set of 24 bread wheat varieties with diverse phenotypes supported by their large-scale phenotypic variation in agronomic and RSA traits^14,15^. We initially analyzed the dataset to identify expression variation of potential transcripts or genes involved in RSA and validated by correlation analysis with RSA phenotypes.

## Methods

### Plant material

A panel of 24 historical spring wheat cultivars released in Pakistan was selected for this study. The cultivar name, year of release, and pedigree are given in Table 1. These cultivars selected based on the year of release to represent the cultivated diversity over the course of 110 years.

**Table 1:** List of historical spring wheat cultivars with release year and pedigree.

Name	Year of Release	Pedigree
T9	1911	Landrace
C518	1933	T9/8 A
C217	1944	C 516 X C 591
C271	1957	C230/IP165
Drik	1958	C-271/WILLET-DWARF//SONORA-64
Maxipak-65	1965	PJ/GB55 or PJ62/GB55
Photowar-70	1970	BURT/KENYA//QUETA(L)/3/NAD63
Pari-73	1973	CNO67//SN64/KLRE/3/8156
Pak-81	1981	KVZ/BUHO//KAL/BB
Barani-83	1983	BB/GLL/3/GTO/7 C//BB/CNO67
Chakwal 86	1986	FORLANI/ACC//ANA
Rawal 87	1987	MAYA/MON//KVZ/TRM
Pasban-90	1990	INIA F66/TH.DISTICHUM//INIAF66/3/GENARO T81
Inquilab-91	1991	LR64A/NAI60
Parwaz-94	1994	C271/WT(E)/SN64
Punjab-96	1996	URES/BOW’S
Chakwal-97	1998	BUC’S’/FCT’S’
G.A.2002	2002	DWL5023/SNB//SNB
Seher-2006	2006	CNO67//SN64/KLRE/3/8156
Chakwal-50	2008	ATTILA/3/HUI/CARC//CHEN/CHTO/4/ATTILA
NARC-2009	2009	INQALAB 91*2/TUKURU
Dharabi-2011	2011	HXL-7573/2*BAGULA//PASTOR
Pakistan-13	2013	MEX94.27.1.20/3/Sokoll//Attila/3*BCN
Ujala-2016	2016	KIRITATI/4/2*WEAVER/TSC//WEAVER/3/WEAVER

### Growth and RNA isolation

The seeds of 24 wheat cultivars were surface sterilized using 3% NaOCl and were sown in triplicates in plastic trays containing peat moss. Two weeks after germination (at Zadoks stage 2), seedling leaf and root tissues were collected and subjected to total RNA extraction. RNA extraction was performed using EasyPure Plant RNA Kit (ER301-01) following the instructions provided by manufacturer and quantified using Nanodrop 2000 spectrophotometer (Thermo Fisher Scientific, USA).

### RNA Sequencing and identification of differentially expressed genes

The RNA samples were sequenced from Beijing Genomics Institute (BGI), China. For cDNA synthesis, the oligo (dT) method was used. The 50-bp single-end sequencing libraries were constructed, and BGISEQ-500 platform was used for sequencing using standard protocols. ‘Clean data’ was produced as FastQ data files using SOAPnuke version 2.1.6. Mapping with reference genome of bread wheat^16^ was done using HISAT2 software v 2.2.1^17^. Bowtie software was used for alignment of reference sequence with reads^18^. The reads were then quantified using featureCounts software and differentially expressed genes (DEGs) were identified using DeSEQ. 2 in R v 4.1.1. The threshold value for filtering of DEGs was set at 0.1. All the DEG files were then culminated into a single file used for further analysis^19^. The R codes were used to generate heatmaps directly from the normalized count file^20^, or phenotypic data from the diversity panel was used to calculate correlation values and plot correlation values as heatmaps^20^.

### Phenotyping for agronomic traits and root system architecture

The agronomic traits of the diversity panel were taken from our previous experiment^21^. Briefly, the diversity panel was planted at five locations and important agronomic traits were recorded. The phenotyping for RSA architecture traits has been described in detail^14^. The imaging platform consisting of RhizoVision crown hardware^22^ controlled by RhizoVision Imager software was used for root image acquisition and details have been described previously^14^. The RSA traits included in the study were maximum weight (MaxW), maximum diameter (MaxD), lower root area (LRA), median number of roots (MNR), steep angle frequency (StAF), solidity (S), volume diameter (VD), surface area (SA), network area (NtA), projected area diameter (PAD), surface area diameter (SAD), median angle frequency (MAF), average root orientation (ARO), shallow angle frequency (SAF), depth (D), width to depth ratio (WDR), maximum number of roots (MaxNR), number of root tips (NRT), volume (V), perimeter (P), total root length (TRL), root length diameter (RLD), convex area (CA), average diameter (AD), and median diameter (MD). The correlation between gene expression and various traits including RSA traits, root hair length and density under low and high phosphorous treatments, and yield-related traits was determined using ‘psych’ package in R version 4.2.1.

## Data Records

In total, 130 RNA-seq datasets were generated which are deposited to the SRA repository of NCBI under BioProject PRJNA863398^23^. The gene list of differentially expressed genes (DEGs) from each tissue is submitted to the Gene Expression Omnibus (GEO) repository under accession number GSE235844^24^. The raw count data table from RNAseq data of each tissue was generated and converted to normalized tpm values and deposited at FigShare^20^ under 10.6084/m9.figshare.23292389 and DryAd^19^ under 10.5061/dryad.zs7h44jcs.

The summary statistics of raw reads from leaf and root tissues are given in Supplementary Table 1.

## Technical Validation

RNA quality was initially determined using NanoDrop and samples <1.8 values of OD260/280 and OD260/230 were further processed for RNA integrity. RNA integrity was assessed with Agilent 2100 Bioanalyzer RNA Nano assay (Agilent Technologies, USA). The average RIN values were 7.3 ± 0.5 for leaf samples and 6.9 ± 0.4 for root tissues. The qRT-PCR validation of selected genes was also performed.

### Quality assessment

The aligned FastQ data files were read using SAMTools software and basic statistics are presented in Supplementary Table 1. The average quality score which is ratio between the sum of base qualities and total length was >60 in all cases. The error rate which is mismatches per bases mapped was <0.001.

### Initial analysis of differentially expressed genes

**Fig. 1:**
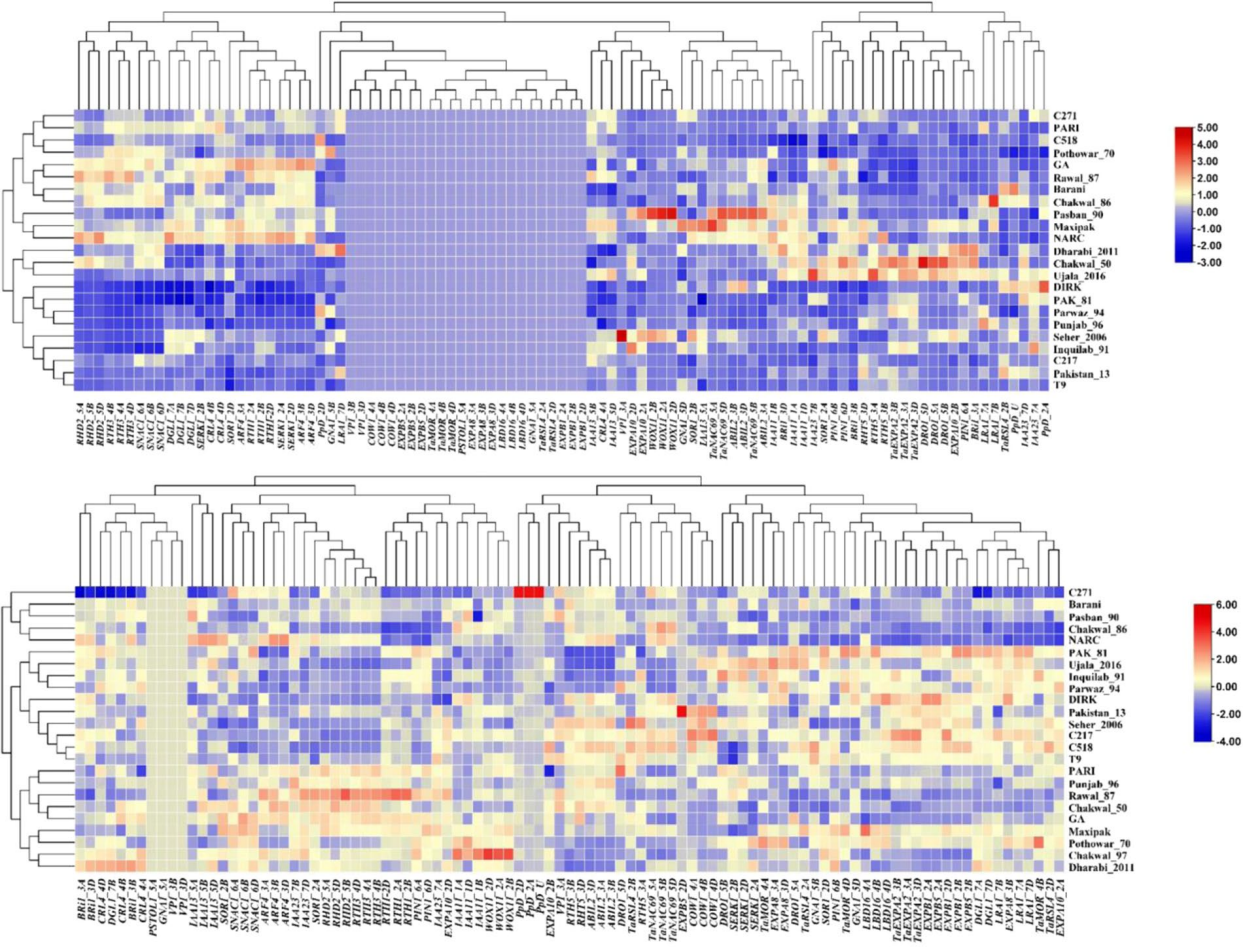
Differential expression of RSA-related genes in (**A**) leaf and (**B**) root tissues of 24 bread wheat cultivars. The original gene names have been used while Traes IDs can be found in the associated excel file available at FigShare under 10.6084/m9.figshare.23292389.

**Fig. 2:**
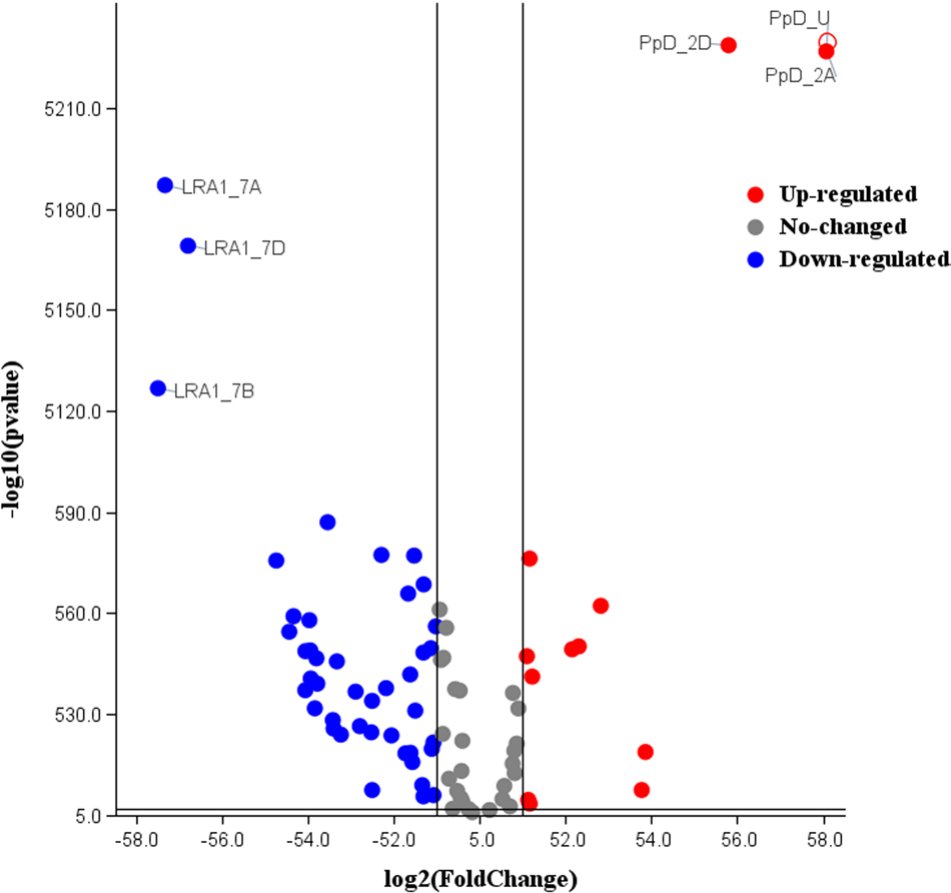
Volcano plot showing up- and down-regulation (leaf vs root) of root-related genes. Only top six genes are labelled.

**Fig. 3:**
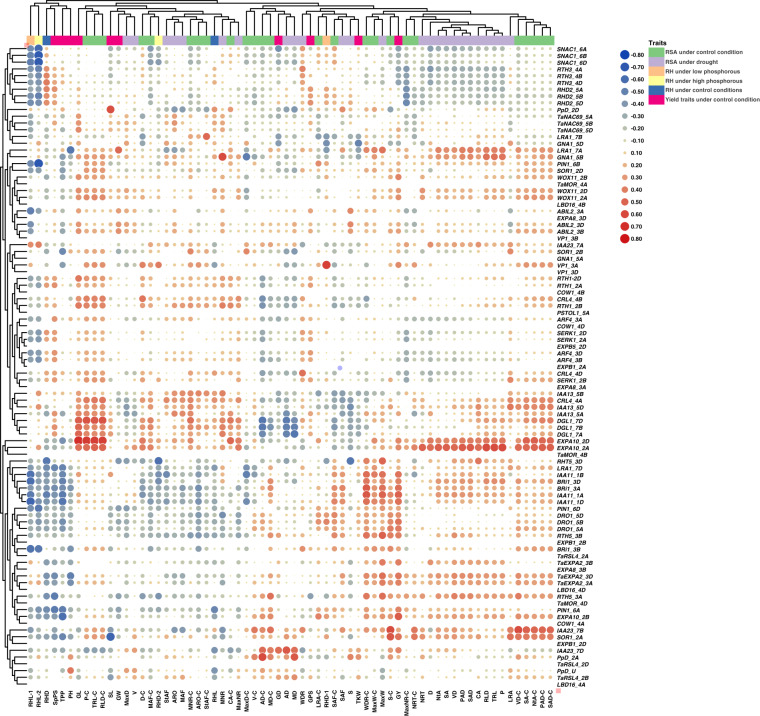
Heatmap showing significant correlations between expression of RSA-related genes in leaf, yield-related traits, and root traits under control and drought stress conditions. The size of the circle explains the extent of correlation. Traits are abbreviated as; Maximum weight (MaxW), Maximum diameter (MaxD), Lower root area (LRA), median number of roots (MNR), steep angle frequency (StAF), solidity (S), volume diameter (VD), surface area (SA), network area (NtA), projected area diameter (PAD), surface area diameter (SAD), median angle frequency (MAF), average root orientation (ARO), shallow angle frequency (SAF), depth (D), width to depth ratio (WDR), maximum number of roots (MaxNR), number of root tips (NRT), volume (V), perimeter (P), total root length (TRL), root length diameter (RLD), convex area (CA), average diameter (AD), median diameter (MD), spikes per spike (SpPS), plant height (PH), tillers per plant (TPP), grain yield (GY), grain length (GL), thousand kernel weight (TKW), grain density (GD), grains per spike (GPS), spike length (SL), and grain weight (GW).

**Fig. 4:**
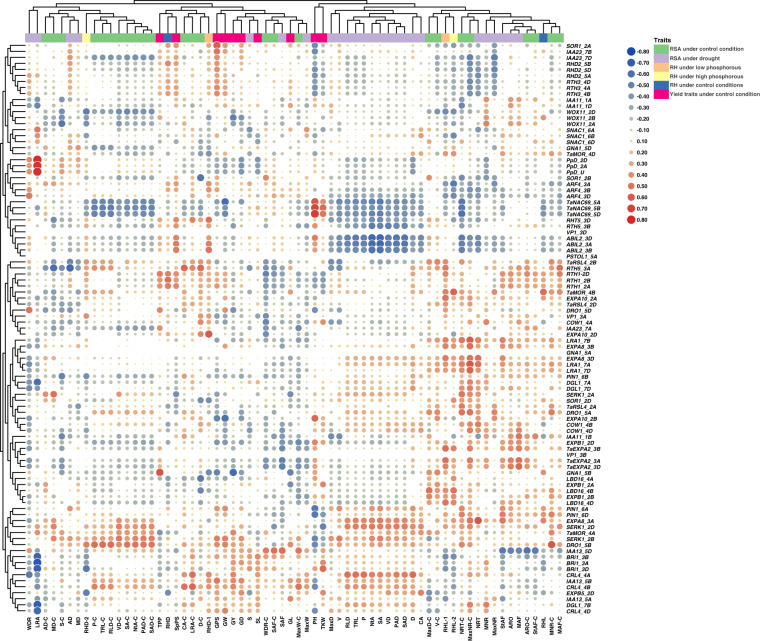
Heatmap showing significant correlations between expression of RSA-related genes in roots, yield-related traits, and root traits under control and drought stress conditions. The size of the circle explains the extent of correlation. Traits are abbreviated as; Maximum weight (MaxW), Maximum diameter (MaxD), Lower root area (LRA), median number of roots (MNR), steep angle frequency (StAF), solidity (S), volume diameter (VD), surface area (SA), network area (NtA), projected area diameter (PAD), surface area diameter (SAD), median angle frequency (MAF), average root orientation (ARO), shallow angle frequency (SAF), depth (D), width to depth ratio (WDR), maximum number of roots (MaxNR), number of root tips (NRT), volume (V), perimeter (P), total root length (TRL), root length diameter (RLD), convex area (CA), average diameter (AD), median diameter (MD), spikes per spike (SpPS), plant height (PH), tillers per plant (TPP), grain yield (GY), grain length (GL), thousand kernel weight (TKW), grain density (GD), grains per spike (GPS), spike length (SL), and grain weight (GW).

The gene list of differentially expressed genes (DEGs) from each tissue is submitted to the Gene Expression Omnibus (GEO) repository under accession number GSE235844. A total of 38 RSA related genes were identified from various cereal species including wheat, rice, maize, and barley from the published literature. The blastn analysis was carried out to identify their A-, B- and D-sub-genome homeologues of those genes. This process identified 95 homeologues in wheat which were then used in the subsequent analyses. The gene IDs, names, description and GO ontology are given in Supplementary Table 2. The GO enrichment analysis was performed using Triticeae-Gene Tribe, a homology database^25^ (Supplementary Table 3). Initially, heatmaps were generated for 95 genes using gene IDs (TraesIDs) for tpm values in leaf (Fig. 1A) and root tissues (Fig. 1B). The expression profiles of the selected genes extracted from the main DEG file using the R code^20^. The heatmaps were generated using TBTools. In leaf tissues, all genes were expressed except *TaMOR, PSTOL1, EXPA8, LBD16, EXPB1, COW1*, and *EXPB5*. No expression of *VP1* gene was observed in leaf tissues in all varieties except Seher-2006 which showed highest expression of *VP1_3A* (Fig. 1A). In contrast to leaf tissues, all genes exhibited differential expression patterns in roots except three homoeologues of *Ppd* gene and D homoeologue of *EXPB5* (Fig. 1B). Volcano plot showing differentially expressed gene across 24 cultivars is presented as Fig. 2. The expression of these genes was differential in across root tissues. Leaf gene expression was found significantly correlated with yield traits (Fig. 3). The differential expression of RSA concerning genes in roots was significantly correlated to all 25 root traits under optimum conditions with some variations (Fig. 4). The dataset and initial analysis proved very effective in culminating the differential gene expression in root tissues and due to the large number of samples, it was possible to associate gene expression data with the phenotypes. In conclusion, the differential expression of these genes in the roots provided a validation of the dataset.

### Supplementary information

Supplementary Table

## Data Availability

FigShare R code used to generate Figs. 3, 4 is available at 10.6084/m9.figshare.23292389 along with the source file. FigShare R code to generate heatmap of any given wheat gene IDs (using TraesIDs) using the normalized count data file is available at 10.6084/m9.figshare.23292389. The code is also available on GitHub repository as: https://github.com/plantbiologyqau/R-code-for-gene-expression-heatmap/tree/main
